# Muscle Regeneration with Intermuscular Adipose Tissue (IMAT) Accumulation Is Modulated by Mechanical Constraints

**DOI:** 10.1371/journal.pone.0144230

**Published:** 2015-12-02

**Authors:** Allan F. Pagano, Rémi Demangel, Thomas Brioche, Elodie Jublanc, Christelle Bertrand-Gaday, Robin Candau, Claude A. Dechesne, Christian Dani, Anne Bonnieu, Guillaume Py, Angèle Chopard

**Affiliations:** 1 Université de Montpellier, INRA, UMR866 Dynamique Musculaire et Métabolisme, F-34060, Montpellier, France; 2 Université Nice-Sophia Antipolis, iBV, CNRS UMR7277, INSERM U1091, 06107, Nice, France; West Virginia University School of Medicine, UNITED STATES

## Abstract

Sports trauma are able to induce muscle injury with fibrosis and accumulation of intermuscular adipose tissue (IMAT), which affect muscle function. This study was designed to investigate whether hypoactivity would influence IMAT accumulation in regenerating mouse skeletal muscle using the glycerol model of muscle regeneration. The animals were immediately hindlimb unloaded for 21 days after glycerol injection into the tibialis anterior (TA) muscle. Muscle fiber and adipocyte cross-sectional area (CSA) and IMAT accumulation were determined by histomorphometric analysis. Adipogenesis during regenerative processes was examined using RT-qPCR and Western blot quantification. Twenty-one days of hindlimb unloading resulted in decreases of 38% and 50.6% in the muscle weight/body weight ratio and CSA, respectively, in soleus muscle. Glycerol injection into TA induced IMAT accumulation, reaching 3% of control normal-loading muscle area. This IMAT accumulation was largely inhibited in unloading conditions (0.09%) and concomitant with a marked reduction in perilipin and FABP4 protein content, two key markers of mature adipocytes. Induction of PPARγ and C/EBPα mRNA, two markers of adipogenesis, was also decreased. Furthermore, the protein expression of PDGFRα, a cell surface marker of fibro/adipogenic progenitors, was much lower in regenerating TA from the unloaded group. Exposure of regenerating muscle to hypoactivity severely reduces IMAT development and accumulation. These results provide new insight into the mechanisms regulating IMAT development in skeletal muscle and highlight the importance of taking into account the level of mechanical constraint imposed on skeletal muscle during the regeneration processes.

## Introduction

The capacity of skeletal muscle to regenerate is a key parameter of its plasticity. A wide variety of stress can induce muscle injuries, including sport traumas, prolonged blood flow disruption or even muscle diseases. After injury, skeletal muscle is able to regenerate through various and high coordinated stages including degeneration, inflammation, and regeneration process [1]. These steps include recruitment of satellite cells (SCs), which are localized between the sarcolemma and the basal lamina of myofibers [2]. Indeed, it is now well known that quiescent satellite cells proliferate, migrate and differentiate into mature myofibers to regenerate injured muscle tissue [3–5]. Numerous studies have already shown that hindlimb unloading (HU), commonly used to mimic hypoactivity and also microgravity [6], induces a loss in SC content and mitotic activity, which disturbs muscle regeneration by reducing growth of the newly formed myofibers [7–9].

The research literature also indicates that abnormal fibrosis and intermuscular adipose tissue (IMAT) accumulation occur, particularly when early regeneration processes are altered, and that this in turn alters muscle function. IMAT is defined as adipocyte accumulation between muscle cells and beneath the muscle fascia, and it should not be confused with intra-myocellular triglyceride accumulation [10]. Studies have shown that impaired macrophage function is linked to poor muscle regeneration and IMAT accumulation after freeze-induced [11], ischemic [12, 13], notexin-induced [14] and cardiotoxin-induced [15] injury.

In these regeneration models, little or no IMAT accumulation is naturally observed. Although IMAT does not occur naturally in rodent skeletal muscles, a skeletal muscle regeneration model with IMAT accumulation was developed in rabbit by Kawai et al. [16] and was later used in mice in several studies [17–20]. This regeneration model consists of injecting glycerol into skeletal muscle, and Dani’s group was the first to present a detailed characterization of the “glycerol approach” [21]. The model has been used in several studies to investigate IMAT development and its related adipogenic processes and, more recently, to better characterize muscle-resident adipocyte precursors [19, 20, 22]. To our knowledge, the study of Lukjanenko et al. [22] has been the only one to provide a detailed characterization of some of the cellular responses related to this regeneration model in comparison with the more classic cardiotoxin model. Their study clearly showed that the two models induced similar kinetics of skeletal muscle degeneration and regeneration, but they differed with regard to the adipogenic response amplitude. The glycerol model was therefore associated with more mature adipocytes accumulation.

Recently, studies have highlighted the growing importance of muscle-resident mesenchymal stem cells in the regeneration process of skeletal muscle [23, 24]. In particular, fibro/adipogenic progenitors (FAPs), which are mainly positive for the cell surface marker platelet-derived growth factor receptor alpha (PDGFRα or CD140a), play an important role in efficient regeneration. In a healthy but damaged muscle, FAPs proliferate, phagocytize necrotic debris, and increase the proliferation of SCs without differentiating into adipocytes [19, 25]. In muscle disuse or pathological conditions, such as Duchenne muscular dystrophy, FAPs proliferate and differentiate into adipose and/or fibrous tissue [26, 27]. Parallel to the decrease in SC content [28, 29], FAPs in this case lead to an accumulation of IMAT [10, 20].

Although it is clear that disturbed regeneration promotes IMAT accumulation and impaired skeletal muscle function [30], there is a lack of data evaluating the effects of prolonged hypoactivity on IMAT accumulation during regeneration processes. In a closed context, the study of Jarvinen and Lehto [31] conducted in rats showed that immobilization following contusion injury limited the size of the connective tissue area formed within the injury site. One might therefore ask whether a period of hypoactivity would modulate IMAT development in a regeneration model characterized by IMAT development. Our study was designed to investigate and characterize the effects of prolonged hypoactivity induced by hindlimb unloading on IMAT development and accumulation in the glycerol model of muscle regeneration. Rodent HU was set up immediately after glycerol injection to ensure hypoactivity during the entire period of the regeneration process. Our results clearly showed that IMAT accumulation in regenerating muscle was substantially lowered with unloading conditions, with a subsequent decrease in FAP recruitment.

## Materials and Methods

### Ethics statement

This study was approved by the Committee on the Ethics of Animal Experiments of Languedoc Roussillon in accordance with the guidelines from the French National Research Council for the Care and Use of Laboratory Animals (CEEA-LR-14002). All efforts were made to minimize animal suffering.

### Animals

Experiments were carried out on 6-month-old C57BL6J/CBA female mice (n = 36; mean body mass = 25.4g ±0.44) from our own stock. Animals were maintained on a 12h/12h light–dark cycle and provided with food and water ad libitum. Experiments were performed at 22°C.

### Experimental procedures and muscle sampling

Experimental procedures were performed under anesthesia using isoflurane inhalation. In accordance with the study of Pisani et al. [21], the mice were injected with 25μl of 50% v/v glycerol in the right tibialis anterior (TAg) and with saline solution in the contralateral tibialis anterior (TA). Two experimental groups were formed: hindlimb-unloaded (HU, n = 6) and control (CTL, n = 6), for 21 days. Tail-suspended experiments were conducted with suspension cages and the protocol used in other studies [32, 33]. At the end of the 21 days of unloading or control conditions, *soleus* (SOL) and *extensor digitorum longus* (EDL) hindlimb muscles were rapidly dissected out and immediately frozen in isopentane cooled with liquid nitrogen and then stored at -80°C. For the first set of experiments, TAg and TA muscles were also rapidly dissected out and immediately fixed overnight in 4% paraformaldehyde solution at room temperature and then paraffin-embedded. For the second and third set of experiments, TAg and TA from CTL and HU animals were dissected out and rapidly frozen in liquid nitrogen for quantification of mRNA and protein content, as described elsewhere.

### Histology

Transverse serial sections of SOL and EDL muscles (9μm thick) were obtained at -20°C using a cryostat and sections were subjected to hematoxylin-eosin staining for subsequent cross-sectional area (CSA) measurements.

TA and TAg muscles were fixed in 4% neutral-buffered formalin (24h) and paraffin-embedded. The paraffin-embedded tissues were sectioned (3μm thick) every 50μm over the entire muscle depth, and sections were processed by hematoxylin/eosin/saffron (H/E/S) staining. Stained slides were digitalized with the NanoZoomer slide scanner with a x40 objective (Hamamatsu) for subsequent muscle fibers and adipocytes CSA evaluation. Images were analyzed with ImageJ^®^ (1.46r version) software to measure the CSA.

### Immunohistochemistry

The immunohistochemistry protocol was globally performed as previously described [34]. Briefly, mouse colon (positive control) and TAg muscle sections were deparaffinized, rehydrated, and incubated for antigen retrieval in EDTA buffer at 100°C for 10 min. Sections were incubated in 0.3% H_2_O_2_ for 20 min and endogenous biotins were blocked using the Avidin-Biotin Blocking kit (Vector Laboratories, CliniSciences). Nonspecific antibody binding was blocked by incubation with TBS containing 20% normal goat serum for 30 min at RT. Sections were then incubated ON at 4°C with rabbit anti-PDGFRα diluted at 1:250 or non-specific rabbit IgG (Vector Laboratories, CliniSciences) at the same concentration. Antibody binding was revealed by the streptavidin/biotin-peroxydase complex method using ABC Vectastain kit and the peroxidase substrate DAB (Vector Laboratories, Clinisciences).

### RNA extraction and real-time polymerase chain reaction (RT-qPCR)

Total RNAs were isolated from homogenate muscle samples using the RNeasy Fibrous Tissue Mini Kit following the manufacturer’s instructions (Qiagen). RNA concentration was determined by spectrophotometric analysis (Eppendorf AG, Hamburg, Germany), and integrity was checked by the OD_260nm_/OD_280nm_ absorption ratio (>1.7). Reverse transcription reaction was performed with 2μg of total RNA using the RevertAid First Strand cDNA Synthesis kit (Thermo Scientific) according to the manufacturer’s instructions. qPCR analysis was performed in a MiniOpticon detection system (Bio-Rad, Hercules, CA) with 10μL of KAPA SYBR Fast Universal Readymix (CliniSciences), 300nM of both forward and reverse primers, 2μL of diluted cDNA template and water to a final volume of 20μL. The forward and reverse primers used to amplify genes are listed in Table 1. All PCRs were performed in duplicate using the following cycle parameters: 30s at 98°C, 40 cycles of 1s at 95°C and 15s at 60°C. Relative mRNA levels were normalized to ribosomal protein S9 (rpS9) and cyclophilin A housekeeping gene levels, which were unaffected by treatments. Results are expressed using the comparative cycle threshold (C_T_). The relative changes in the level of a specific gene were calculated with the ΔΔC_T_ formula.

**Table 1:** Real-time PCR primers.

Gene	Forward	Reverse	Amplicon size
C/EBPα	GACCAGAAAGCTGAGTTGTGAG	CCACAAAGCCCAGAAACCTA	69 bp
C/EBPβ	CTCCAGGTAGGGGCTGAAGT	TTTAGACCCATGGAAGTGGC	150 bp
Cyclophilin A	TTCCTCCTTTCACAGAATTATTCCA	CCGCCAGTGCCATTATGG	75 bp
MyoD	AGCACTACAGTGGCGACTCA	GGCCGCTGTAATCCATCAT	75 bp
myogenin	ACAGGCCTTGCTCAGCTC	CGCTGTGGGAGTTGCATT	102 bp
Pax7	GCTACCAGTACAGCCAGTATG	GTCACTAAGCATGGGTAGATG	328 bp
PDGFRα	AAGACCTGGGCAAGAGGAAC	GAACCTGTCTCGATGGCACT	67 bp
PPARγ	GTGCCAGTTTCGATCCGTAGA	GGCCAGCATCGTGTAGATGA	142 bp
rpS9	CGGCCCGGGAGCTGTTGACG	CTGCTTGCGGACCCTAATGT	247 bp

### Protein isolation and Western blotting

The Western blot protocol was performed as previously described [35]. Briefly, muscle samples were homogenized in 10 volumes of lysis buffer (50mM Tris–HCl pH7.5, 150mM NaCl, 1mM EGTA, 100mM NaF, 5mM Na_3_VO_4_, 1% Triton X-100, 40mM β-glycerophosphate and protease inhibitor mixture (P8340; Sigma-Aldrich)) and centrifuged at 10.000g for 10 min (4°C). 60μg of protein extracts were loaded on SDS–polyacrylamide gels before electrophoretic transfer onto a nitrocellulose membrane (Bio-Rad). After transfer, membranes were blocked with 50mM Tris-HCl pH7.5, 150mM NaCl, and 0.1% Tween 20 (TBS-T) containing 5% skimmed milk or BSA and incubated overnight at 4°C with primary antibodies. Membranes were incubated for 1 h with a peroxidase-conjugated secondary antibody. Immunoblots were revealed using a Pierce ECL kit (32106; Thermo Scientific) and proteins were visualized by enhanced chemiluminescence and quantified with ImageJ^®^ (1.46r version) software. β-actin was used as a loading control.

### Antibodies

Anti-PDGFRα (#3174), anti-perilipin (#9349), anti-FABP4 (#3544) and anti-adiponectin (#2789) primary antibodies were purchased from Cell Signaling and used at 1:500. Anti-β-actin (sc-81178) primary antibody was purchased from Santa Cruz and used at 1:2000. Anti-mouse (NA931V) and anti-rabbit (NA934V) HRP-conjugated secondary antibodies were purchased from GE Healthcare Life Science and used at 1:3000.

### Statistics

All values are expressed as mean ± SEM and the significance level was set as p<0.05. Differences between the two groups were evaluated for significance using the unpaired Student t-test or the Mann-Whitney test when data deviated from a normal distribution. When more than two simultaneous comparisons were made, a two-way ANOVA was employed to compare data (unloading and glycerol-injection factors). When a significant effect was indicated, a Fisher significant difference post hoc test was performed.

## Results

### Body mass, muscle mass, and fiber cross-sectional areas following 21 days of hindlimb unloading

**Fig 1:**
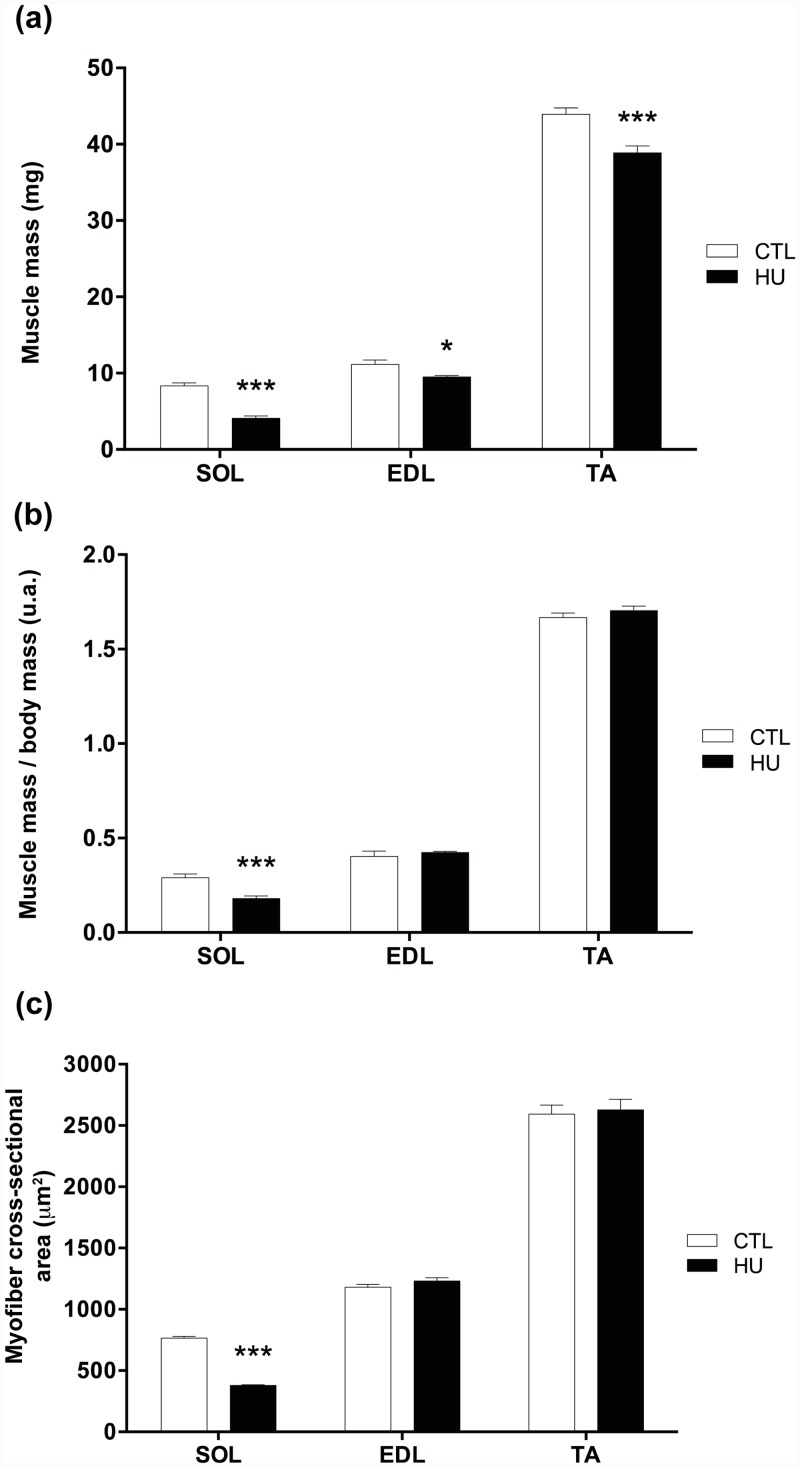
Effects of hindlimb unloading on muscle mass and fiber cross-sectional areas. Soleus (SOL), extensor digitorum longus (EDL), tibialis anterior (TA) skeletal muscle mass from control (CTL) and hindlimb unloading (HU) mice **(a)**. SOL, EDL, and TA mass normalized by body mass from CTL and HU mice **(b)**. Myofiber cross-sectional area (CSA) in μm² of SOL, EDL, and TA skeletal muscles from CTL and HU mice **(c)**. * *p*<0.05 *vs* control group, and *** *p*<0.001 *vs* control group.

Body mass was significantly changed after 21 days of HU (24.57g at day 0 and 22.81g at day 21, p<0.001), whereas no difference was found in control conditions (26.13g at day 0 and 26.47g at day 21). SOL muscle mass, expressed in either absolute values or values normalized by body mass, decreased significantly after HU (-50.8% and -38% respectively, p<0.001, Fig 1a and 1b) compared with CTL. EDL and TA muscle mass decreased significantly after HU, but only when expressed in absolute values (-15.1%, p<0.05, and -11.5%, p<0.001, respectively, Fig 1a and 1b). The mean CSA of SOL fibers was significantly reduced after HU compared with CTL (-50.6%, p<0.001, Fig 1c). However, no significant differences were detected for the mean CSA of EDL and TA muscle fibers (Fig 1c). Thus, our results are in agreement with the literature, which has shown major atrophy in SOL, a postural muscle, following 21 days of HU [6, 36].

### Hindlimb unloading disturbs regeneration in glycerol-injected tibialis anterior

**Fig 2:**
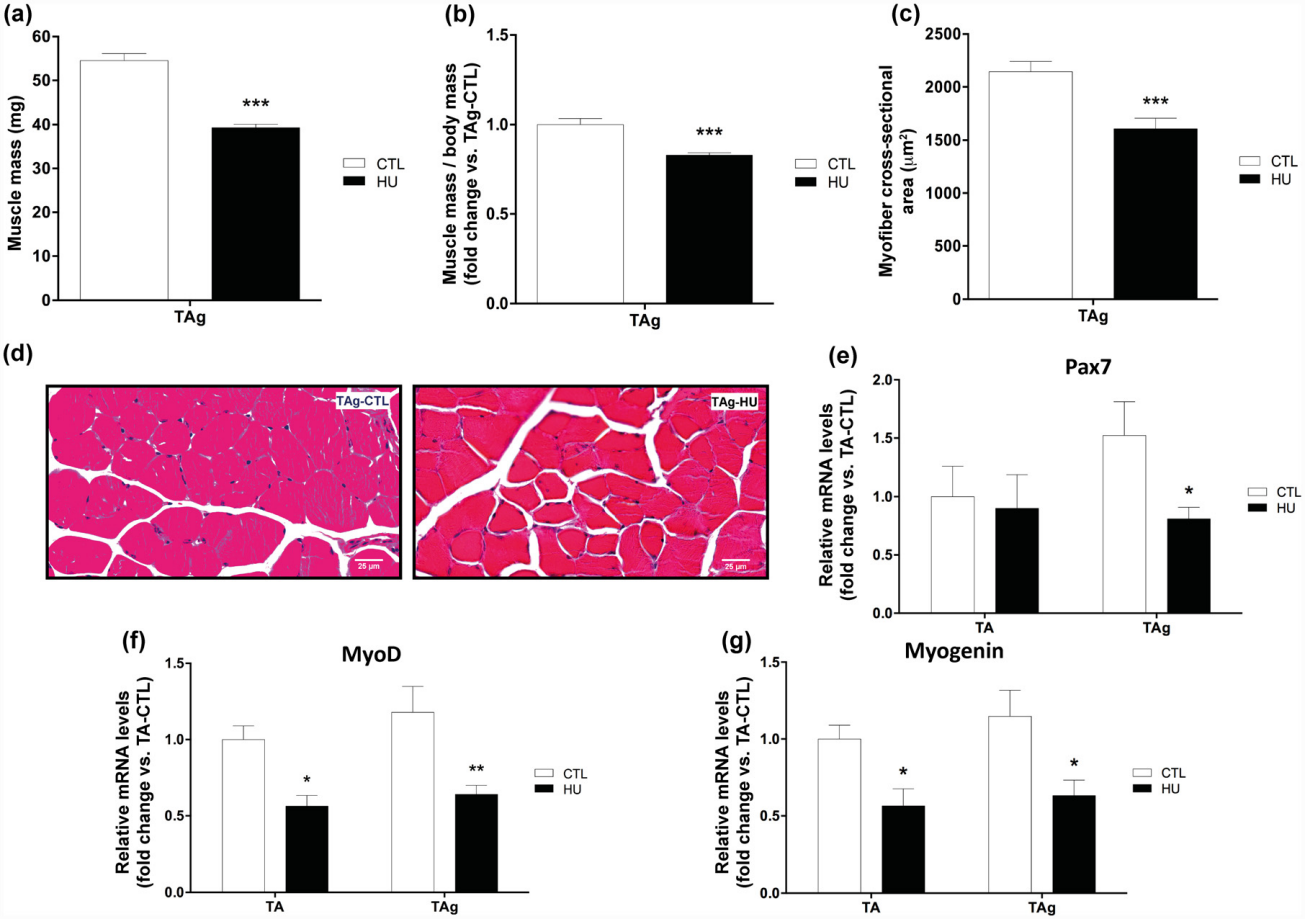
Effects of hindlimb unloading and glycerol injection on tibialis anterior muscle. Muscle mass **(a)**, muscle mass/body mass **(b)** and cross-sectional area (CSA) **(c)** of glycerol-injected tibialis anterior (TAg) from control (CTL) and hindlimb unloading (HU) mice. Representative histological transverse paraffin-embedded muscle sections, stained with hematoxylin-eosin-saffron from TAg of each experimental group (CTL and HU) **(d)**. Pax7 **(e)**, MyoD **(f)** and myogenin **(g)** mRNA levels of TA and TAg from CTL and HU mice. * *p*<0.05 *vs* control group, ** p<0.01 *vs* control group, *** *p*<0.001 *vs* control group (unloading effect).

Glycerol-injected tibialis anterior (TAg) muscle mass, expressed in either absolute values or values normalized by body mass, decreased significantly after HU (-27.87% and -17.1% respectively, p<0.001, Fig 2a and 2b) compared with TAg-CTL. In parallel, the mean CSA of TAg-HU fibers *versus* TAg-CTL was significantly reduced (-25.1%, p<0.001, Fig 2c and 2d). This suggests that hindlimb unloading affects skeletal muscle regeneration and thereby reduces myofiber size and mass recovery. We further examined the mRNA induction of key genes implicated in the muscle regeneration processes and myogenesis in TA and TAg of the CTL and HU groups. mRNA induction of Pax7, a marker of quiescent and proliferative SCs in adult mice, decreased only in TAg-HU compared with TAg-CTL (-46.9%, p<0.05, Fig 2e). Levels of MyoD and myogenin mRNA, two markers of activated and differentiation-engaged myoblasts, respectively, were reduced significantly in TA-HU *versus* TA-CTL (-43.7% and -43.5% respectively, p<0.05, Fig 2f and 2g). A similar decrease in magnitude was also observed in TAg-HU *versus* TAg-CTL (-45.7%, p<0.01 and -44.85%, p<0.05, respectively, for MyoD and myogenin, Fig 2f and 2g). Taken together, these results based on mRNA analysis could indicate that hindlimb unloading alone did not alter the number of SCs but did decrease their mitotic activity. However, when HU was associated with a regenerating context, we found a decrease in the SC pool and mitotic activity, which thus decreased the subsequent regrowth and myofiber size recovery.

### Hypoactivity inhibits IMAT accumulation in regenerating TA muscle

**Fig 3:**
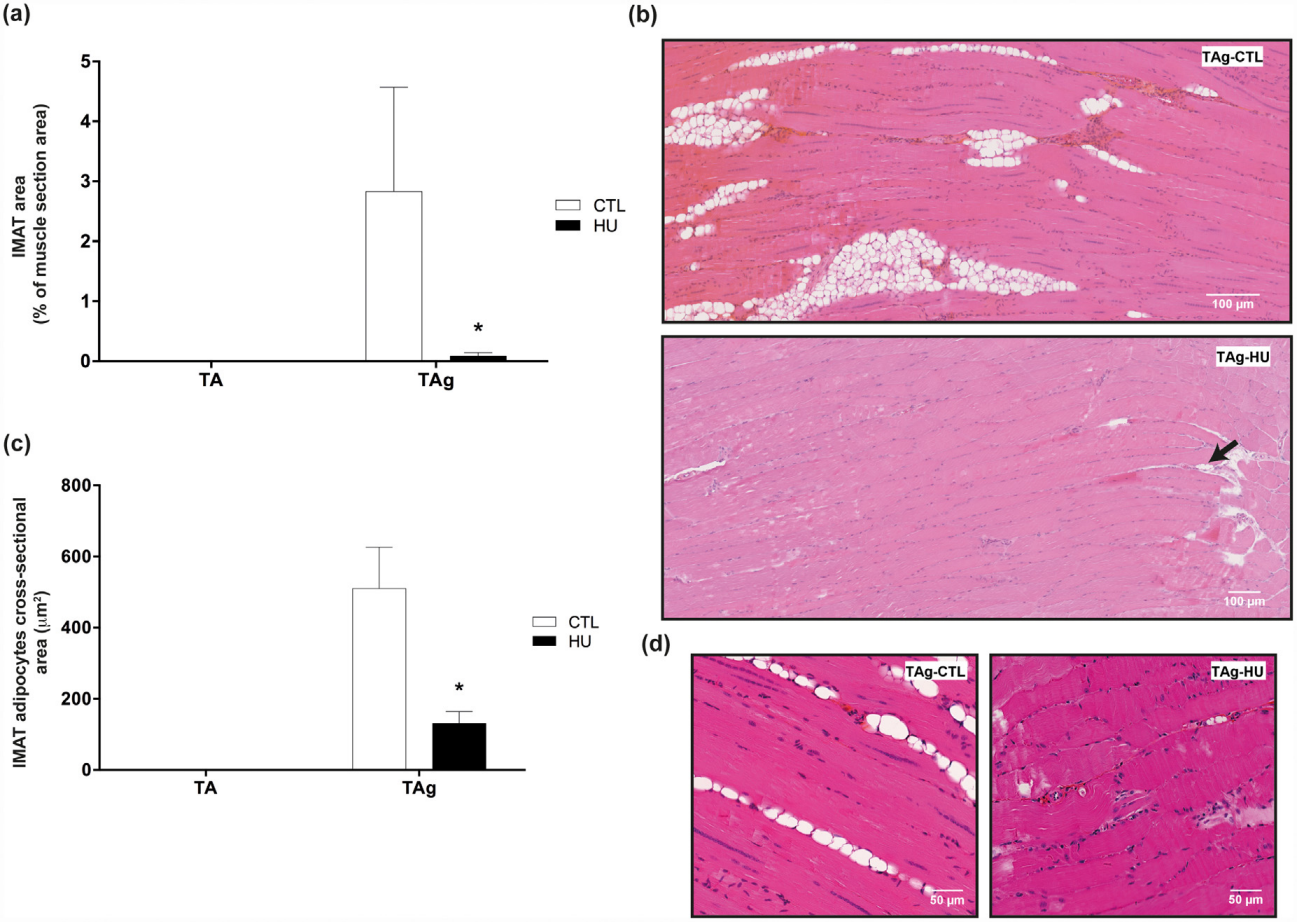
Effects of hindlimb unloading on IMAT development and accumulation. Intermuscular adipose tissue (IMAT) area in percentage of total muscle section area **(a)** and IMAT adipocyte cross-sectional area (CSA) in μm² **(c)** of saline-injected (TA) and glycerol-injected (TAg) tibialis anterior from control (CTL) and hindlimb unloading (HU) mice. * *p*<0.05 *vs* control group. Representative histological longitudinal paraffin-embedded muscle sections, stained with hematoxylin-eosin-saffron from TAg of each experimental group (CTL and HU) **(b, d)**.

It is well known that the glycerol model of muscle regeneration induces IMAT development during muscle regeneration. In our study, we confirmed the presence of IMAT in the TA injected with glycerol of normal-loaded animals (TAg-CTL). The area occupied by IMAT 21 days after glycerol injection reached a mean 2.83% of the total muscle CSA, whereas TA-CTL showed no histological sign of IMAT (Fig 3a and 3b). Thus, as previously described in the study of Dani’s group [21], it seems that the muscle microenvironment created by glycerol injection may favor adipogenesis from resident and/or recruited precursors, unlike what occurs in other regeneration models.

Conversely, unloading almost completely prevented IMAT development in TAg: the area occupied by IMAT was almost nil (0.09%, p<0.05) in TAg-HU (Fig 3a and 3b). Moreover, CSA morphometric analysis of the adipocytes from IMAT showed a decrease in TAg-HU compared with TAg-CTL (130μm^2^ and 510μm^2^, respectively, p<0.05, Fig 3c and 3d).

**Fig 4:**
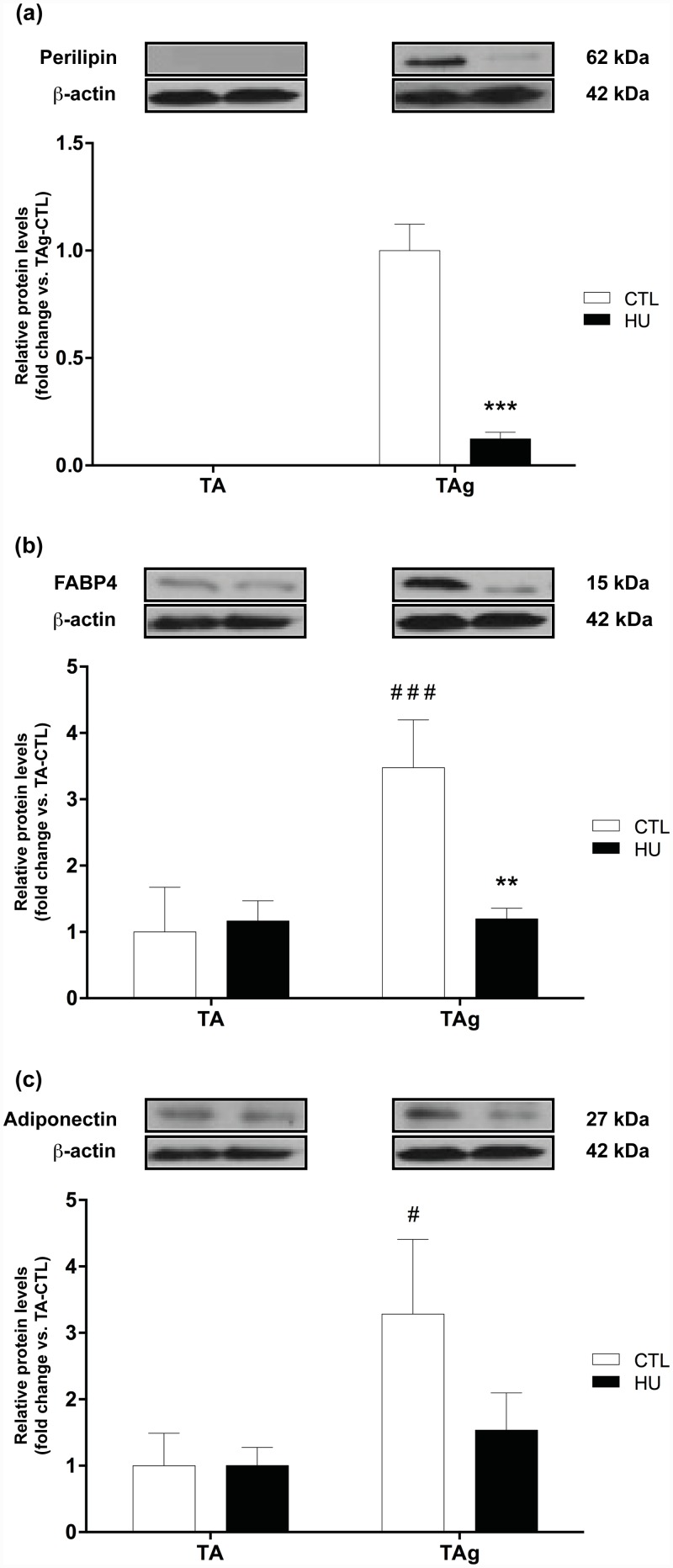
Protein expression levels of mature adipocyte markers. Perilipin **(a)**, FABP4 **(b)** and adiponectin **(c)** protein content of saline-injected (TA) and glycerol-injected (TAg) tibialis anterior from control (CTL) and hindlimb unloading (HU) mice. ** *p*<0.01 *vs* control group, *** *p*<0.001 *vs* control group (unloading effect). # *p*<0.05 *vs* TA-CTL, ### *p*<0.001 *vs* TA-CTL (glycerol-injection effect).

To further confirm the effects of hypoactivity on IMAT development, we quantified the expression of perilipin, fatty acid binding protein 4 (FABP4) and adiponectin, three markers of mature adipocytes. Concerning perilipin, the adipocyte lipid droplet protein marker, no signal was detected for the saline-injected TA, whereas its expression was clear in TAg, as expected (Fig 4a). In line with our previous results, perilipin expression in TAg was considerably decreased by HU (-87.7%, p<0.001, Fig 4a). The expression level of FABP4, a cytoplasmic fatty acid chaperone, increased in TAg-CTL compared with TA-CTL (+246.7%, p<0.001) and returned to baseline level in TAg-HU (p<0.01, Fig 4b). The expression level of adiponectin, an adipokine, increased in TAg-CTL compared with TA-CTL (+228.2%, p<0.05), whereas this difference was not observed between TAg-HU and TA-HU (Fig 4c). All together, these results were in agreement with our histological findings and confirmed that HU reduces IMAT accumulation in this model of muscle regeneration.

### Hypoactivity decreases the adipogenesis processes in regenerating muscles

After determining that unloading was able to inhibit IMAT development in the glycerol regeneration model, we investigated the underlying mechanisms that led to the decrease in IMAT development in HU conditions. We first analyzed the mRNA induction and protein expression of PDGFRα, the cell surface marker of FAPs, and then we investigated the induction and/or expression of different markers involved in adipogenesis.

**Fig 5:**
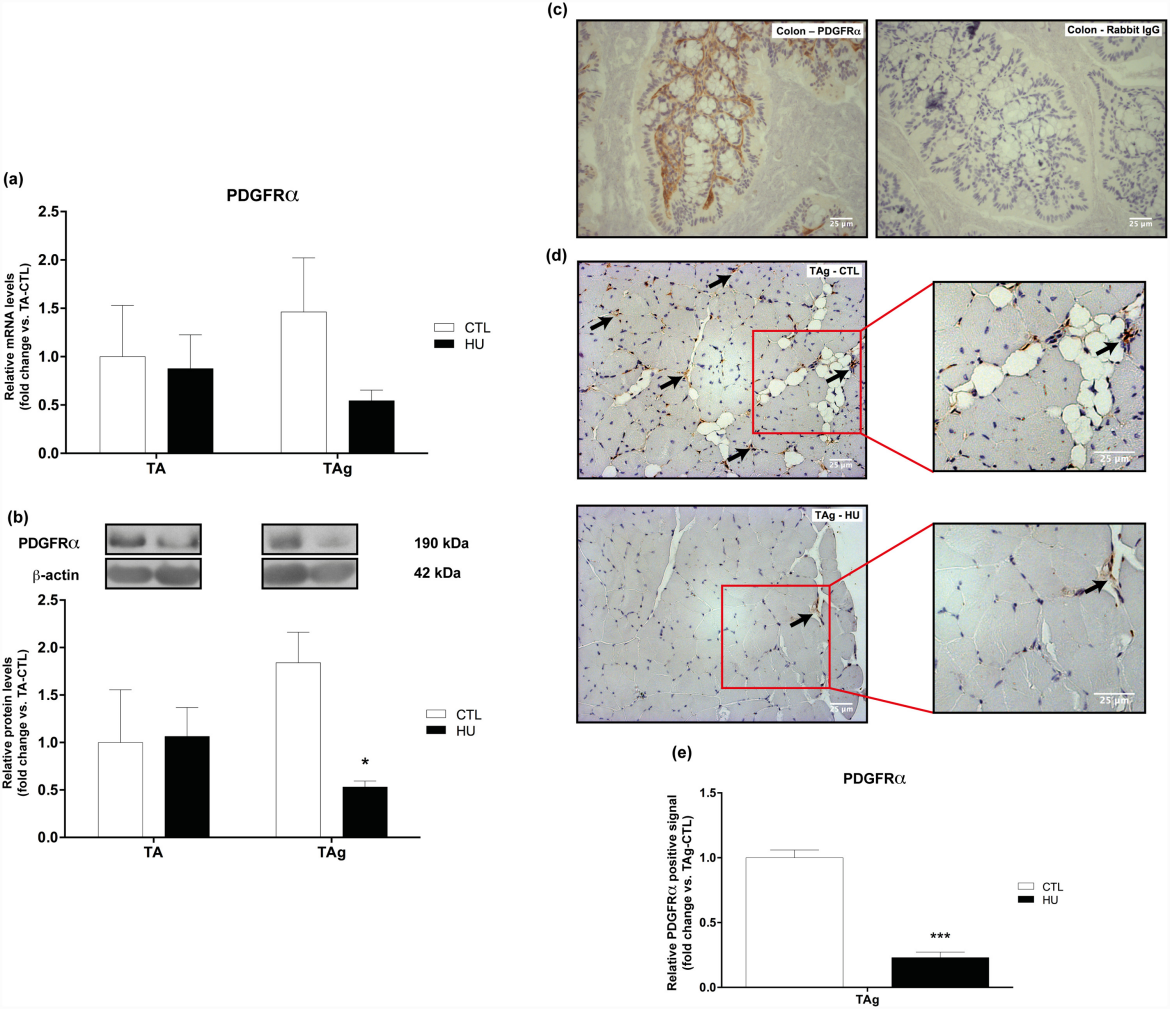
Expression levels of the FAP cell surface marker PDGFRα. PDGFRα mRNA **(a)** and protein **(b)** content of saline-injected (TA) and glycerol-injected (TAg) tibialis anterior from control (CTL) and hindlimb unloading (HU) mice. * *p*<0.05 *vs* control group (unloading effect). Representative images **(c, d)** and quantification **(e)** of immunohistochemical analysis of PDGFRα-positive cells in mouse colon positive control **(c)** and glycerol-injected (TAg) tibialis anterior from control (CTL) and hindlimb unloading (HU) mice **(d)**. *** p<0.001 *vs* control group.

**Fig 6:**
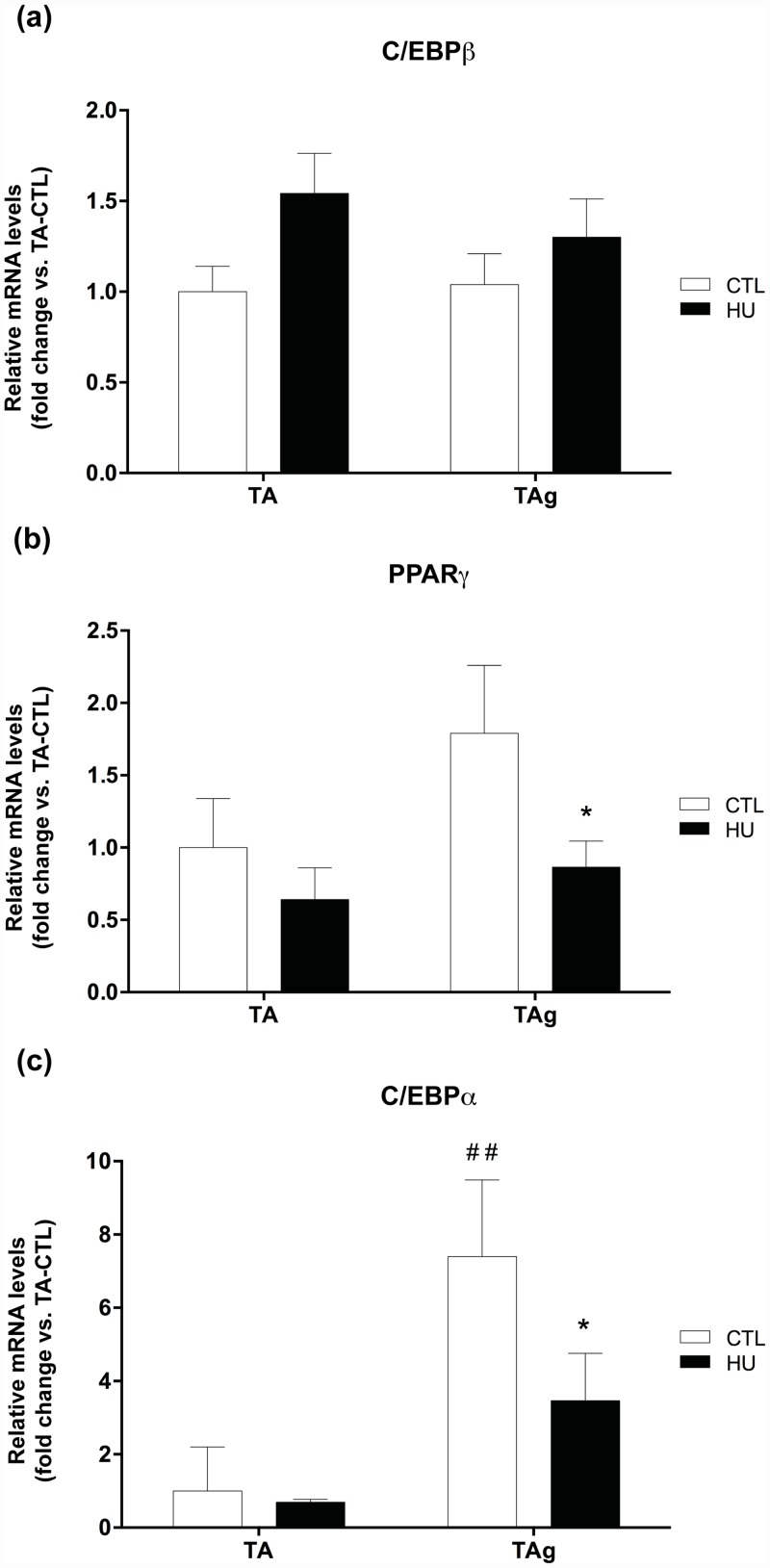
Changes in mRNA induction of adipogenesis markers. C/EBPβ **(a)**, PPARγ **(b)** and C/EBPα **(c)** mRNA levels of saline-injected (TA) and glycerol-injected (TAg) tibialis anterior from control (CTL) and hindlimb unloading (HU) mice. * *p*<0.05 *vs* control group (unloading effect). ## *p*<0.01 *vs* TA-CTL (glycerol-injection effect).

Firstly, we found no differences in PDGFRα protein expression or mRNA induction for saline-injected TA or TAg-CTL conditions (Fig 5a and 5b). The last confirming the return to basal values for PDGFRα marker 21 days after injury reported in the study of Lukjanenko et al. [22]. However, the protein content of PDGFRα was drastically lowered in TAg-HU *versus* TAg-CTL (-71.1%, p<0.05, Fig 5b) and this result was confirmed by our immunohistochemical analysis (-77%, p<0.001, Fig 5d), whereas the respective results for mRNA induction were not significantly different (Fig 5a). Antibody specificity was checked using mouse colon section as a positive control [37] (Fig 5c). As hypothesized, no change was observed in the induction of CCAAT/enhancer-binding protein β (C/EBPβ) mRNA, a transcription factor implicated in an early step in adipogenesis (Fig 6a). However, mRNA induction of peroxisome proliferator-activated receptor-γ (PPARγ) and CCAAT/enhancer binding protein α (C/EBPα), two transcriptional factors implicated in the later steps of adipogenesis, was lower in TAg-HU *versus* TAg-CTL (-51.6% and -53.2%, respectively, p<0.05, Fig 6b and 6c). Furthermore, we observed an approximately 7-fold higher induction of C/EBPα mRNA in TAg-CTL compared with TA-CTL (p<0.01, Fig 6c).

## Discussion

The main objective of this study was to characterize the effects of prolonged hypoactivity on IMAT development in regenerating muscles. For that purpose, we used the well-described skeletal muscle regeneration model of glycerol injection, known to lead to IMAT formation. Our study shows for the first time that hypoactivity almost completely prevents IMAT accumulation.

Although IMAT may be a variable part of healthy human skeletal muscles, its increase and accumulation are synonymous with muscle dysfunction, deconditioning, and even perturbed regeneration [27, 30, 38]. The fat infiltration of muscle remains understudied and little is known about whether hypoactivity can lower or conversely enhance IMAT accumulation in pathological or regenerating skeletal muscle.

In our experiment, TAg muscles exhibited adiposity 21 days after glycerol injection and this result was strengthened by the increase in protein expression of several mature adipocyte markers: perilipin, FABP4 and adiponectin. Moreover, we analyzed the expression of the major transcriptional factors implicated in adipogenesis and found a significant induction of C/EBPα mRNA in TAg-CTL compared with TA-CTL. However, no difference in C/EBPβ and PPARγ mRNA induction was observed, although these two transcriptional factors are known to participate in adipogenesis in a phase earlier than C/EBPα, which appears to be up regulated at the end of the adipogenesis process [39]. This is in accordance with our present study: 21 days after glycerol injection adipogenesis was largely accomplished and the early adipogenic transcription factors had returned to basal values [22].

We further characterized the effects of prolonged hypoactivity on the confirmed IMAT accumulation. For this purpose, we used the well-characterized hindlimb suspension model and our study revealed for the first time an almost complete inhibition of adipogenesis in regenerating muscles induced by the glycerol model submitted to hypoactivity over the entire length of the regeneration period. Our histological examination of TAg-CTL and TAg-HU was performed over the entire depth of the muscle and we clearly observed the inhibition of adipogenesis in unloading conditions. This histological result was further supported by the quantification of perilipin, FABP4 and adiponectin expression, which considerably decreased following HU.

Even though the precise mechanisms leading to IMAT formation in the glycerol model are still unclear, previous studies have described the respective roles of multipotent stem cells such as muscle-derived FAPs, pericytes, and side population cells giving rise to adipogenic precursors [26, 40–43]. Recently, a few groups have identified muscle mesenchymal progenitors with the immunophenotype CD31-CD45-SM/C-2.6-PDGFRα+, which contribute to fat cell formation in skeletal muscle [20, 43, 44]. PDGFRα has also been used very recently in human to isolate muscle mesenchymal progenitors, which are equivalent to the mouse FAPs [27, 45]. We used this FAP cell surface marker in the present study and found a marked decrease in its protein expression following HU. Our study thus indicates that hypoactivity is able to decrease PDGFRα-positive FAPs, which represent 98% of PDGFRα-positive cells in regenerating muscle [19]. Moreover, as reported by Uezumi et al. [20], only the PDGFRα-positive cells can differentiate into adipocytes in glycerol-injected muscles. We further observed reduced levels of the later adipogenic transcription factors PPARγ and C/EBPα. We did not detect any differences for C/EBPβ, certainly due to its earlier implication in adipogenesis [39].

Interestingly, FAPs are also known to promote skeletal muscle regeneration after injury in healthy muscle [19, 25]. Heredia et al. [25] showed that FAP proliferation and macrophage-like activity are essential to the regeneration process. In this context, decreased FAP proliferation could result in a decreased number of adipocytes, as well as impair the regenerative kinetics [25]. Skeletal muscle regenerative capacity is mainly dependent on the activation of SCs, which are finely controlled by the myogenic regulatory factors. Quiescent and proliferative SCs express the paired box transcription factor (Pax7) and its inactivation leads to a severe depletion of these muscle myogenic stem cells [46]. In regenerating muscle, the proliferating process is triggered by the expression of myoblast determination protein 1 (MyoD) and myogenic factor 5 (Myf5) [47, 48]. Once differentiation is initiated, myogenin appears to be implicated first, and then muscle-specific regulatory factor 4 (Mrf4) is activated during the maturation phase [49, 50]. In our study, we first described a decrease in MyoD and myogenin mRNA induction in TA-HU concomitant with no significant effect on Pax7. These results are in accordance with studies demonstrating that HU does not necessarily alter the number of SCs in fast-twitch skeletal muscle, probably indicating no SC loss by apoptosis [51], but that it does alter SC mitotic activity [7, 9, 51, 52]. We then observed a decrease in Pax7, MyoD, and myogenin mRNA induction, parallel to the decrease in muscle mass and CSA in TAg-HU. Taken together, these results confirm that reducing mechanical constraints throughout muscle regeneration disturbs SC-mediated regeneration and thus delays myofiber size recovery, as indicated in the study of Matsuba et al. [53]. Nevertheless, the study of Mozdziak et al. [8] showed that the hindlimb unloading condition does not decrease but could even enhance the mitotic activity of SCs and probably that of non-muscle cells in the earlier stages of regeneration. However, once new myofibers are formed, their growth capacity is altered. Our results suggest that HU may alter regrowth after regeneration, but we cannot exclude the hypothesis that HU may enhance early regeneration processes, thereby inhibiting IMAT occurrence and further disturbing muscle regrowth. Clearly, additional studies are warranted to elucidate the early events related to mesenchymal non-muscle cells and their implication in both the regeneration process and adipogenesis during unloading conditions.

Currently, we do not know the exact underlying mechanisms leading to the inhibition of muscle adipogenesis in regenerating muscles under unloading conditions. As highlighted in the literature, hindlimb unloading appears to be a proinflammatory situation with macrophage infiltration [54–58]. Interestingly, recent studies have reported the critical role of inflammation and the immune system in muscle regeneration [59–62], and it appears that both macrophage shift and activity are essentials in this process. In addition, the study of Lukjanenko et al. [22] revealed that the glycerol injury model exhibits a disrupted inflammatory response compared with the cardiotoxin-induced injury model. Further studies are needed to elucidate the underlying mechanisms of unloading-induced inhibition of skeletal muscle IMAT development and accumulation and especially the effects of an inflammatory response on mesenchymal stem cells.

In conclusion, our study reports for the first time an almost complete inhibition of IMAT development in regenerating muscles under hypoactivity conditions. We found a decreased response of mesenchymal-derived precursor FAPs (PDGFRα^+^), which could explain the decrease in IMAT development in the present model. Hypoactivity seems to locally create a favorable environment leading to a decrease in PDGFRα positive cells.

These observations shed new light on the mechanisms that regulate IMAT development in skeletal muscle and highlight the importance of taking into account the level of mechanical constraint imposed on skeletal muscle during regeneration processes. Our findings point in the same direction as those reported by Jarvinen and Lehto [31] concerning immobilization, which was found to mediate a decrease in fibrotic area after gastrocnemius injury in rats.

In the one hand, our results suggest that a rest period with reduced mechanical constraints might be needed immediately after injury to prevent IMAT accumulation. However, our study also shows that the regrowth of skeletal muscle fibers is impaired under hypoactivity, which highlights the importance of applying mechanical constraints as soon as possible after the rest period for the recovery of fiber size. Our present and future studies should contribute to a fuller understanding of IMAT accumulation and the establishment of rehabilitation guidelines for human muscle injuries.

## Supporting Information

S1 Checklist ARRIVE Checklist. (PDF)
